# Dynamic Control of Synaptic Adhesion and Organizing Molecules in Synaptic Plasticity

**DOI:** 10.1155/2017/6526151

**Published:** 2017-01-31

**Authors:** Gabby Rudenko

**Affiliations:** Department of Pharmacology and Toxicology, Sealy Center for Structural Biology and Molecular Biophysics, University of Texas Medical Branch, 301 University Boulevard Rm. 5.114B, Galveston, TX 77555, USA

## Abstract

Synapses play a critical role in establishing and maintaining neural circuits, permitting targeted information transfer throughout the brain. A large portfolio of synaptic adhesion/organizing molecules (SAMs) exists in the mammalian brain involved in synapse development and maintenance. SAMs bind protein partners, forming* trans*-complexes spanning the synaptic cleft or* cis*-complexes attached to the same synaptic membrane. SAMs play key roles in cell adhesion and in organizing protein interaction networks; they can also provide mechanisms of recognition, generate scaffolds onto which partners can dock, and likely take part in signaling processes as well. SAMs are regulated through a portfolio of different mechanisms that affect their protein levels, precise localization, stability, and the availability of their partners at synapses. Interaction of SAMs with their partners can further be strengthened or weakened through alternative splicing, competing protein partners, ectodomain shedding, or astrocytically secreted factors. Given that numerous SAMs appear altered by synaptic activity, in vivo, these molecules may be used to dynamically scale up or scale down synaptic communication. Many SAMs, including neurexins, neuroligins, cadherins, and contactins, are now implicated in neuropsychiatric and neurodevelopmental diseases, such as autism spectrum disorder, schizophrenia, and bipolar disorder and studying their molecular mechanisms holds promise for developing novel therapeutics.

## 1. Synapses

It is estimated that there are more than one hundred billion neurons in the human brain, connected to one another by hundreds of trillions of contact points called synapses. These synaptic connections wire select neurons into functional neuronal circuits, enabling the brain to process and transfer information. Each synapse consists of a patch of “*presynaptic membrane*” from one neuron (typically an axon terminus) adhered to a patch of “*postsynaptic membrane*” from a second connecting neuron (typically a dendrite), and the space between them called the “*synaptic cleft.*” At chemical synapses, which comprise the vast majority of synapses in the brain, action potentials (i.e., electrical activity) from the presynaptic neuron trigger the release of neurotransmitters into the synaptic cleft, provoking molecular and cellular responses in the postsynaptic neuron in a process referred to as “*synaptic activity.*” The presynaptic side of the synapse hosts the molecular machinery needed to release and recycle synaptic vesicles containing these neurotransmitters. The postsynaptic side of the synapse responds to the released vesicle contents via receptors and channels and triggers downstream cellular responses. It is still not well understood how neurons wire together into specific circuits and, in particular, how correct synaptic connections are established and maintained over time. Remarkably, synaptic connections are dynamic and can change. In response to synaptic activity, they undergo structural and functional alterations as part of a process called “*synaptic plasticity.*” Synaptic plasticity can involve changes to the molecular components present at a synapse, the location of these components at a synapse, the efficiency with which a synapse can communicate, and even whether a synapse is maintained or completely disappears; for excellent recent reviews see [1–3]. Mechanisms of synaptic plasticity are widely believed to be involved in long term memory [1–3]. Alterations at synapses have commonly been monitored through two important phenomena:* long term potentiation* (LTP) and* long term depression* (LTD), processes that cause an increase or decrease in synaptic strength, respectively (as gauged by the electrical output produced by the postsynaptic neuron in response to synaptic stimulation). LTP and LTD are also thought to be involved in memory and learning. Synapses, thus, through their role in mediating connections between neurons and their ability to change through mechanisms of synaptic plasticity play an essential role in proper brain function.

## 2. Protein Networks at Synapses and Their Relation to Disease

Synapses contain a staggering number of proteins. Extensive proteomics studies and review of the literature estimate that there are ~1,900 to more than ~2,700 proteins localized at synapses [4–6]. The synaptic proteins identified include ones involved in exocytosis and recycling of synaptic vesicles, receptors for different neurotransmitters, ion channels, extracellular matrix proteins, cell adhesion molecules, cytoskeletal proteins, scaffolding proteins, membrane transporters, GTPases, phosphatases, and molecules involved in protein degradation. However, where the exact boundary of a synapse lies is vague (i.e., where it starts and stops), so scientists have typically relied on the ability of a protein to be co-isolated with synaptosomal membrane fractions and/or microscopy to designate a synaptic identity. Also it is not known which of these many different proteins are found at a particular synapse or how their distribution and expression level varies over the different synapse types.

More than a decade ago, it was suggested that defects at synapses would underlie many neurodevelopmental and neuropsychiatric diseases [7]. Hundreds of genes are now implicated in diseases like schizophrenia, autism spectrum disorder, and other behavioral and cognitive disorders, and many of them indeed encode synaptic proteins ([8–11]; https://sfari.org/resources/sfari-gene). For this reason, the term “synaptopathies” is increasingly used to refer to neurodevelopmental, neurodegenerative, and neuropsychiatric disorders that involve the disruption of synaptic proteins [12, 13]. Given the myriad of proteins found at synapses, synapses can best be viewed as large protein interaction networks that are plastic and change in response to synaptic activity; in addition, disruption of these synaptic networks contributes to the pathology of many neurological disorders.

## 3. Synaptic Adhesion Molecules in the Synaptic Cleft

**Figure 1:**
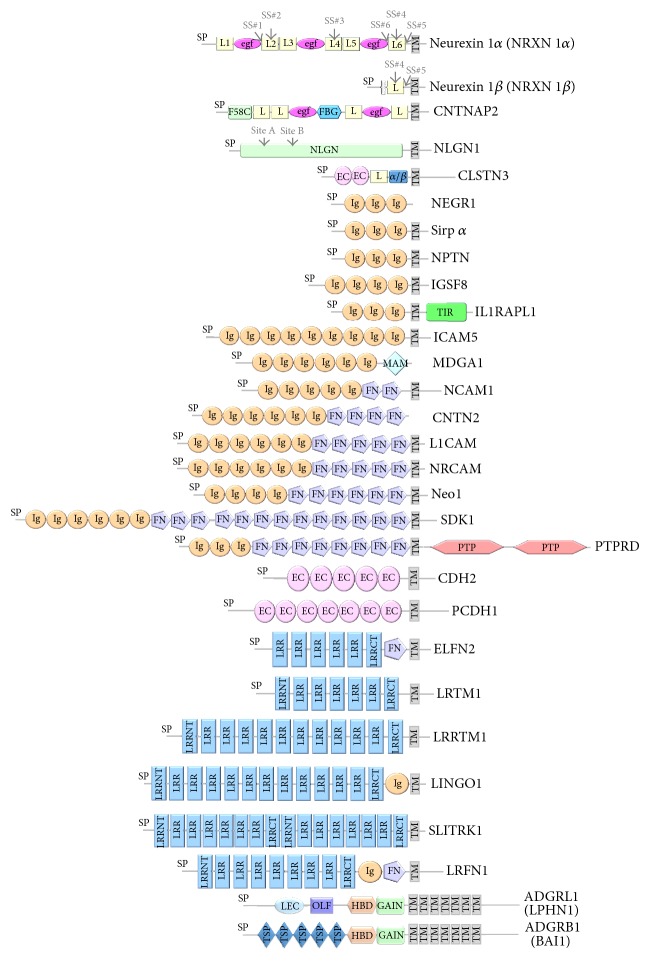
SAMs. Prominent families of SAMs with putative synaptic localization and function are shown. Prototypes used to depict the domain organization are indicated. From top to bottom the following is listed: neurexin 1*α* and neurexin 1*β* (NRXN1*α* and NRXN1*β*; neurexins); CNTNAP2 (contactin associated protein-like); NLGN1 (neuroligins); CLSTN3 (calsyntenins); NEGR1 (Iglons which include NEGR1, NTM, LSAMP, and OPCML); Sirp *α* (signal regulatory proteins); NPTN (neuroplastin); IGSF8 (immunoglobulin superfamily); IL1RAPL1 (interleukin 1 receptor accessory protein-like); ICAM5 (intercellular adhesion molecules); MDGA1 (MAM domain containing glycosylphosphatidylinositol anchor); NCAM1 (neural cell adhesion molecules); CNTN2 (contactins); L1CAM (L1 cell adhesion molecules); NRCAM (neuronal cell adhesion molecules); Neo1 (neogenin); SDK1 (sidekick cell adhesion molecules); PTPRD (protein tyrosine phosphatase receptor types D, F, and S); CDH2 (cadherins); PCDH1 (protocadherins); ELFN2 (extracellular leucine rich repeat and fibronectin type III domain containing); LRTM1 (leucine rich repeats and transmembrane domains); LRRTM1 (leucine rich repeat transmembrane neuronal); LINGO1 (leucine rich repeat and Ig domain containing); SLITRK1 (SLIT and NTRK-like family member); LRFN1 (leucine rich repeat and fibronectin type III domain containing); ADGRL1 (adhesion G protein-coupled receptor type L; previously known as latrophilins); ADGRB1 (adhesion G protein-coupled receptor type B, previously known as brain-specific angiogenesis inhibitor). Several large polymorphic families including the ephrin receptors, integrins, and plexins are not shown. The domain abbreviations used in the text are for laminin G or laminin G/neurexin/sex hormone binding globulin or LNS domains (L); epidermal growth factor repeat (EGF); coagulation factor 5/8 type C (F58C); fibrinogen-like (FBG); extracellular cadherin (EC); alpha/beta (*α*/*β*); immunoglobulin (Ig); Toll/Il-1 receptor homology (TIR); meprin, A-5 protein, receptor protein tyrosine phosphatase mu (MAM); fibronectin type 3 (FN), protein tyrosine phosphatase (PTP); leucine rich repeat (LRR), N-terminal leucine rich repeat (LRRNT); C-terminal leucine rich repeat (LRRCT); galactose binding lectin domain (LEC); olfactomedin-like domain (OLF); hormone binding domain (HBD); GPCR-autoproteolysis inducing (GAIN); thrombospondin (TSP). Other abbreviations are signal peptide (SP) and transmembrane segment (TM). Alternative splice insert sites are indicted for the SAMs NRXN1*α*, NRXN1*β*, and NLGN1, as they are referred to in the text.

One large class of proteins found at synapses contains the so-called* synaptic adhesion molecules* (SAMs). Some SAMs are also called “*synaptic organizers*” because they coordinate protein interaction networks. SAMs are tethered to the presynaptic or postsynaptic membrane by a transmembrane segment or GPI anchor and extend their extracellular domains into the synaptic cleft. Their ectodomains range in size from relatively small (just a single domain) to very large (10 or more domains). Examples of prominent SAM families with putative adhesive or organizing functions are shown in Figure 1. Some families of SAMs are composed of members sharing very similar domain compositions (e.g., cadherins with five EC domains), while other families contain members that have very divergent extracellular regions (e.g., the immunoglobulin super family, IGSFs). Strikingly, many of the well-known SAM families use a limited number of modules to compose their extracellular domain, alternating, for example, immunoglobulin, fibronectin type 3, cadherin, laminin G, and leucine rich repeat domains in different ways (Figure 1).

How many SAMs are found in the human brain? Large databases cataloguing synaptic proteins such as SynaptomeDB [6] and SynProt [5] provide a tremendous starting point to derive an estimate. However, these databases are still incomplete. For instance, validated SAMs like calsyntenins and sidekicks (Figure 1) are absent in SynaptomeDB and SynProt, likely because they have evaded detection in the mass spectrometry-based proteomic studies around which these databases were heavily constructed [14, 15]. On the other hand, a multitude of potential SAMs have been identified by proteomic and genomic studies, for which precise function and synaptic localization have yet to be validated. In addition, many large families containing validated SAMs also contain many members that are as yet uncharacterized with respect to their adhesive and organizing roles and synaptic localization, so it is not known if these also function as SAMs. Therefore, though likely in the hundreds or even thousands (if considering splice variants), the number of* bona fide* SAMs in the human brain is not accurately known.

**Figure 2:**
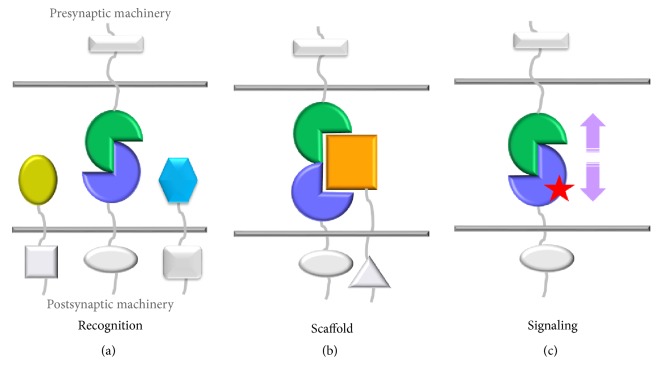
SAM function and mechanisms. SAMs can recruit and organize protein interaction networks in the synaptic cleft by (a) generating mechanisms to recognize specific SAM partners, but not others by binding through direct interactions; (b) binding other SAMs to generate a scaffold onto which a third protein can dock and this mechanism also supports the binding of SAMs through indirect interactions; (c) binding a partner and inducing a signaling event, for example, through (allo)steric mechanisms.

What are the functions of SAMs? Traditionally, SAMs have been evaluated according to their adhesive function, assessed typically via their ability to aggregate cells in cell-based assays, copurify with synaptosomal membrane fractions, and localize to synapses in electron microscopy images. However, in recent years, a dramatically expanded and more nuanced view has emerged for the function of these molecules. It has become clear that SAMs can support a bewilderingly large number of different kinds of protein interactions at synapses. Through their extracellular domains, SAMs can bind protein partners in the synaptic cleft. They can form strict homophilic interactions with identical molecules, semihomophilic interactions with related family members, and/or heterophilic interactions with members of other adhesion molecule families. Via their cytoplasmic tails, SAMs can bind partners intracellularly, integrating into the presynaptic and/or postsynaptic machinery. When SAMs bind partners tethered to the opposing membrane, a “*trans*-complex” is formed that generates a macromolecular bridge spanning the synaptic cleft. At the same time, the components recruited to their cytoplasmic tails from the presynaptic and postsynaptic machineries align. These* trans*-complexes support the adhesive function observed for SAMs in cell-based assays. However, SAMs are increasingly being recognized for essential roles beyond simply adhering the presynaptic and postsynaptic membranes together. They can play a role in neuron-neuron recognition and generate scaffolds onto which additional proteins can bind, and some SAMs likely signal to the presynaptic and/or postsynaptic membranes (Figure 2). For example, some SAMs can bind partners tethered to the same membrane in a side-by-side fashion forming a “*cis*-complex.” Such* cis-*protein assemblies are often regulatory in nature, altering, binding, or forming an essential precomplex onto which a third partner can dock to yield the final* trans*-synaptic bridge, for example, MDGA1 binding to neuroligin 2 (NLGN2) [16, 17], the CNTNAP2-CNTN2 tripartite system [18], and SALM4 binding to SALM3 which inhibits* trans*-synaptic SALM3-LAR adhesion [19]. SAMs can also interact with nonadhesion molecules like receptors, channels, and secreted factors in the synaptic cleft working to recruit and organize synaptic protein networks (or “*synaptic interactomes*”). These different modes of interaction enable SAMs to play important roles in synapse specification, not only organizing the protein interaction networks at a synapse, but also specifying functional properties, for example, by altering presynaptic release probabilities, and/or neurotransmitter receptor and channel properties; for an excellent review see [20].

A vast body of experimental work has demonstrated that SAMs play a key role in promoting the formation, development, maturation, stabilization, and eventual elimination of synapses. Through their roles in forming and maintaining synaptic connections, SAMs therefore intimately impact the wiring of neurons into circuits. The monumental task to identify, characterize, and validate each of the many SAMs and their family members has become increasingly pressing with the discovery that so many of these molecules are implicated in neurodevelopment and neuropsychiatric diseases, for example, the neurexins, neuroligins, LRRTMs, CNTNAPs, contactins, cadherins, and protocadherins (Figure 1; for recent reviews see [20–26]). So while initially recognized purely for their ability to adhere cells together, the functions of SAMs are now recognized to be much more broad, nuanced, and subject to complex regulation.

## 4. Plastic Interactions within the Synaptic Cleft

**Figure 3:**
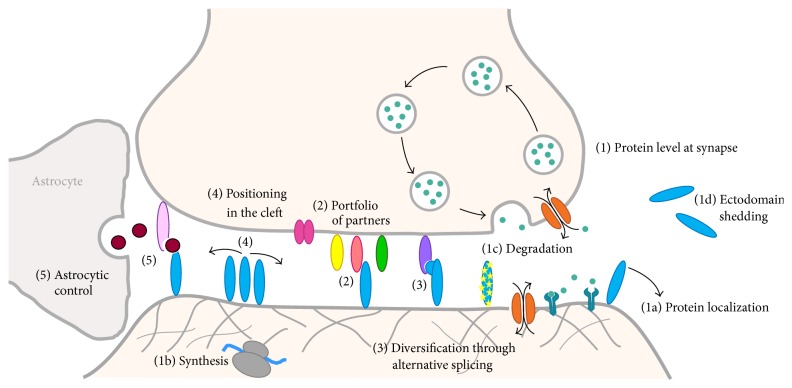
SAMs can contribute to synaptic plasticity. SAM function can be regulated by synaptic activity through different processes. Protein levels can change (1) as a result of altered localization targeting a protein to or away from the synaptic membrane surface (1a), protein synthesis (1b), protein degradation (1c), and ectodomain shedding (1d). The availability of members within a broad portfolio of potential partners can be altered (2). SAMs can be diversified through alternative splicing (3). SAMs can be repositioned in the synaptic cleft (4). Protein interactions supported by SAMs can be modulated by astrocytic factors (5). Details are as discussed in the text.

SAMs are strategically positioned to contribute to synaptic plasticity, given that they can alter synapse structure and function through their ability to sculpt and regulate synaptic protein interaction networks. Below we highlight several important mechanisms that have come to light that regulate SAMs, their diversity, and their functions in a synaptic activity-dependent way. We further present supporting examples to illustrate the general themes (Figure 3).

### 4.1. Alteration of SAM Protein Levels in the Synaptic Cleft

It has long been held that synaptic protein abundance is implicated in synaptic plasticity. In particular, altering the abundance of a specific SAM at a synapse could fundamentally impact the development, maintenance, and ultimate elimination of that synapse. A number of studies have used quantitative proteomics of synaptosomal fractions to correlate synaptic protein abundance (including those of SAMs) to events implicated in synaptic plasticity, for example, the long term synaptic adaptions that accompany the administration of drugs of abuse. Repeated morphine administration robustly downregulated CNTN1, L1CAM, neurocan, and OPCML in striatal presynaptic fractions [27], while in a second study neurexin, NCAM, and NTM protein levels decreased more than 40% in rat forebrain synaptosomal fractions, though in this case OPCML protein levels were unaltered [28]. Importantly, these studies showed that the abundance of synaptic proteins was altered in a highly selectively way. Of 175 proteins that could be identified proteomically, only 30 were robustly and consistently altered by morphine treatment (i.e., 17%), indicating that the SAMs that were altered represented highly significant changes [27]. In other studies, experience dependent plasticity induced in animals by trimming their whiskers to cause sensory deprivation resulted in ~20% to ~30% lower levels for the SAMs Pcdh1, ICAM5, Plexin-A1, and Lphn 3 in juvenile mice (a period where synaptogenesis peaks) [29]. Also in this latter study, the protein abundance was only very selectively altered; only a small number of proteins were affected which included specific SAMs, while 95% of the 7000 tentative synaptic proteins examined showed no significant changes [29]. The above proteomic studies signify that the protein abundance of SAMs can change in response to events triggering synaptic plasticity. However, several caveats exist. These proteomic approaches offer only a global view of protein abundance, profiling changes in protein levels averaged over a large, heterogeneous population of synapses pooled together from many different kinds of neurons and supporting glial cells. In addition, only those proteins that are technically accessible were monitored, that is, only those proteins which were extracted in sufficiently abundant quantities to enable their detection and analysis by mass spectrometric methods [30].

How do protein levels for a specific SAM change in response to synaptic activity at a specific, single synapse or just a small subset of select synapses? Several processes have been identified that modulate SAM protein levels at the level of a single synapse, altering synapse morphology and stabilizing (or destabilizing) synaptic strength on a very local scale in response to synaptic activity (see (1a)–(1d) in Figure 3).

- SAMs can accumulate or be depleted from membrane surfaces in the synaptic cleft as a result of altered stability, for example, due to loss of stabilizing partners, recruitment, trafficking, internalization, and/or phosphorylation of cytoplasmic tails. For instance, levels of neurexin 1*β* at the synaptic membrane rise in response to neural activity, apparently due to an increase in stability (or suppressed dynamics) at the synaptic terminal [31]. NLGN1 and NLGN3 have increased surface membrane levels upon chemically induced LTP and decreased levels after LTD as a result of being dynamically exchanged at the postsynaptic membrane through active cytoskeleton transport [32]. In addition, surface expression of NLGN1 is also increased through CAMKII phosphorylation of its cytoplasmic tail in response to synaptic activity [33]. Other SAMs such as OPCML, CNTN1, and cadherins also display decreasing or increasing protein levels in the synaptic cleft in response to synaptic activity as a result of internalization into the cell or mobilization to the synaptic membrane surface [34–37].
- Protein levels can rise in the synaptic cleft as a result of activity-induced expression via local protein synthesis at the synapse (recently reviewed in [38]). For example, expression of LRRTM1 and LRRTM2 (synaptic organizers that induce presynaptic differentiation) increases as a function of synaptic activity because influx of Ca2+ into the postsynaptic neuron following NMDA-receptor activation induces nuclear Ca2+-dependent transcription [39]. *α*-Dystroglycan expression is also upregulated by prolonged increased neuronal activity at inhibitory synapses in the CNS elevating its protein levels [40]. In addition, local translation of DSCAM in dendrites has been shown to be rapidly induced by synaptic activity [41].
- SAM levels can also decrease at synapses as a result of degradation, thereby regulating synapse development and survival. Evidence is building that highly targeted protein degradation takes place at synapses locally and that it can be regulated by synaptic activity (for recent review see [38]). Intriguingly, elegant studies have revealed that the C. elegans SAM, SYG-1, can locally inhibit an E3 ubiquitin ligase complex that tags proteins for degradation, protecting adjacent synapses from elimination [42].
- One particular form of proteolysis, ectodomain shedding, is now widely documented to regulate SAM protein levels in the synaptic cleft. During shedding, the extracellular domain of a SAM is proteolytically released from its transmembrane segment or its GPI anchor that tethers it to the synaptic membrane. Liberating the SAM ectodomain permits the protein interactions and extracellular matrix to be remodeled within the synaptic cleft. Ectodomain shedding is involved in structural as well as functional synaptic plasticity and impacts key processes like LTP and LTD (for recent reviews see [43, 44]). Exactly where the released ectodomains end up is unclear. Do they remain in the synaptic cleft, binding and blocking their normal protein partners from forming trans-synaptic interactions? Or are the shed ectodomains lost from the synaptic cleft, diffusing outwards to affect other neighboring synapses? Alternatively, are they perhaps simply degraded locally?
- Both presynaptic as well as postsynaptic SAMs have been demonstrated to undergo ectodomain shedding in vitro and in vivo. Activity-dependent proteolytic release has been shown for many well-known SAMs, including neuroligins, neurexins, calsyntenins, SIPR*α*, ICAMs, LARs, Slitrks, and nectins, and their release is executed by various proteases including matrix metalloproteases, ADAM proteases, and alpha/gamma-secretases [14, 43, 45–53]. The downstream consequence of ectodomain shedding varies. In the case of the postsynaptic adhesion molecule NLGN1, shedding destabilizes the presynaptic partner neurexin 1*β* at synapses and decreases the presynaptic release probability of synaptic vesicles, thereby depressing synaptic transmission [48, 50]. Ectodomain release of NLGN1 has relevance for disease, because it is promoted by epileptic seizures [50]. Release of the Sirp *α* ectodomain has a completely different consequence, because it promotes synapse maturation [51]. Likewise, ectodomain release of CLSTN1, which is found on the postsynaptic membrane of inhibitory and excitatory synapses, permits the transmembrane stub and Ca2+-binding cytoplasmic domain to be internalized and accumulate in the spine apparatus where it is thought to carry out a role in postsynaptic Ca2+- signaling [54].

Taken together, multiple processes exist that regulate protein levels of specific SAMs in the synaptic cleft of single synapses in response to synaptic activity.

### 4.2. Availability of a Broad Portfolio of Different SAMs Containing Variable, Synergistic, and Competing Partners

It is estimated that there are more than 470 putative cell adhesion molecules in humans [55], although how many of these are expressed in the brain and are synaptic is not known. Nevertheless, a broad portfolio of SAMs has been validated to date and it provides a powerful mechanism to generate a myriad of different possible interactions, some of which can be affected by synaptic activity, thereby contributing to mechanisms of synaptic plasticity (see (2) in Figure 3). Diversity is achieved in several ways. Most SAMs are modular in nature and use a combinatorial approach to build up their extracellular region by alternating different structural modules, for example, Ig domains, FN3 domains, and cadherin EC domains (Figure 1). In addition, most SAM families contain several members that, while sharing a conserved domain structure, vary in amino acid sequence. In some families, individual members are diversified even further through alternative splicing of their mRNA, inserting, deleting, or exchanging anywhere from one to more than a hundred amino acids in the encoded protein. For instance, more than a thousand splice variants have been demonstrated for neurexins (discussed below).

The portfolio of SAMs can be expanded even further on a functional level in two key ways. First, SAMs can assume evolving functions over time, carrying out one function during the early stages of brain development, while connectivities are being formed, and then switching to another function in the mature adult brain. For example, early during synapse development, neuroligins and LRRTMs appear to compensate for one another; however once synapses have formed, neuroligins and LRRTMs affect excitatory synaptic transmission differently [56]. Likewise, during early development cadherins are important for synapse adhesion, stabilization, and synaptogenesis in young neurons; however once mature synapses have formed, they no longer are needed to keep neuronal and synaptic structures in place but appear to play a role in signaling, structural plasticity, and cognitive function [34]. Second, certain SAMs appear to work together synergistically, generating new functions that do not extend to the individual members alone. Case in point, different combinations of protocadherin family members form dimeric* cis*-complexes that oligomerize into larger tetrameric* trans*-complexes; the functional roles of these different species are still being worked out [57, 58]. Members of different families can also interact with each other in a mix-and-match approach. For example, cadherins bind each other to form* trans*-complexes spanning the synaptic cleft, but they also can bind protocadherins side-by-side forming* cis*-complexes [34].

Given such a broad portfolio of SAMs, how different are the proteins functionally or are many of them redundant? The extent to which different SAMs carry out substantially different functions or are redundant is controversial. Some SAMs clearly have discrete and different biological functions. For example, NLGN1 can induce synapse formation in young primary hippocampal cultures, but SynCAM1 cannot [59]. Members of the same SAM family can also have dramatically different roles; NLGN2 is found exclusively at inhibitory synapses, while NLGN1 is found predominantly at excitatory synapses [23]. Likewise, Slitrk1 and Slitrk3 promote excitatory versus inhibitory synapse formation, respectively [60, 61] However, equally so, SAMs can also demonstrate functionally redundant actions. For instance, LRRTM1, LRRTM2, NLGN1, and NLGN3, proteins that increase synapse numbers in vitro, appear functionally redundant because only knockdown of all four proteins together decreases the number of formed synapses significantly [62]. Thus, though many SAMs exist, their exact functional roles and the extent to which these are unique or overlap needs to be further investigated, both alone and in the broader context of the synaptic cleft.

**Figure 4:**
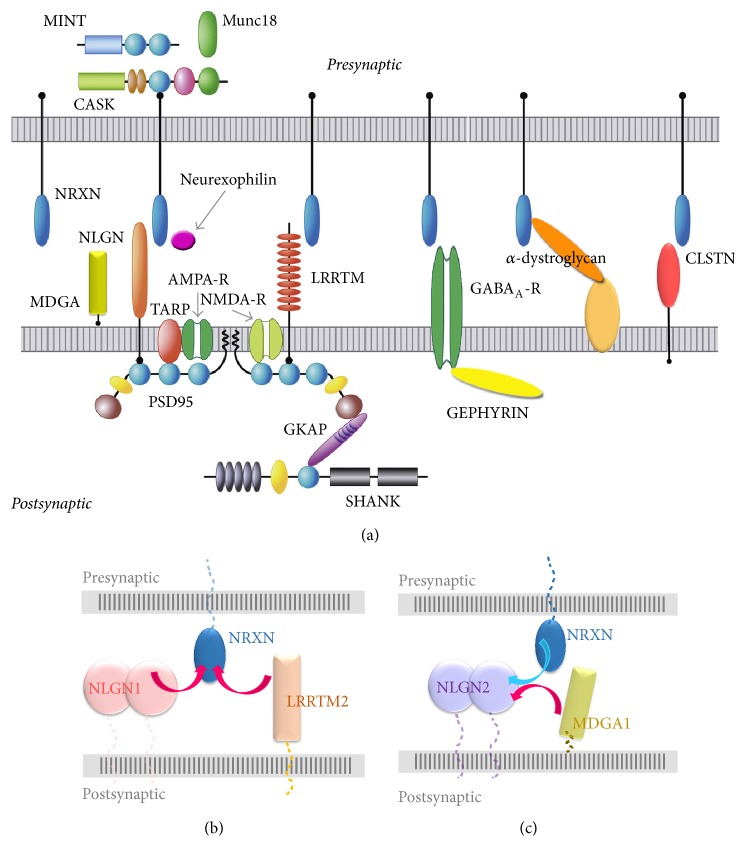
Synaptic protein interaction network coordinated by neurexins. (a) Neurexins (blue ovals) bind many protein partners tethered to the postsynaptic membrane including neuroligins, LRRTMs, *α*-dystroglycan, calsyntenins (CLSTN), and the GABA_A_-receptor, as well as partners that are secreted such as neurexophilins. (b) NLGN1 and LRRTM2 can both bind neurexins at an overlapping binding site generating two competing* trans*-interactions. (c) Neurexins and MDGA1 can both bind NLGN2 at an overlapping binding site generating competing* cis*- and* trans*-interactions.

The power of a broad portfolio of SAMs binding each other and sculpting interactomes within the synaptic cleft is beautifully illustrated by the complex interaction network that has been revealed centered on neurexins. Presynaptically tethered neurexins reach across the synaptic cleft to bind postsynaptic ligands such as the neuroligins, LRRTMs, and *α*-dystroglycan, forming* trans*-synaptic bridges (Figure 4(a)). Neurexins also recruit calsyntenins, though whether this interaction is direct or indirect is debated [14, 63, 64]. At excitatory synapses, neurexins extend across the synaptic cleft to bind LRRTMs or NLGN1 promoting excitatory synapse development [23, 65–67] (Figure 4(b)). Because these postsynaptic partners utilize the same or an overlapping binding surface on neurexin, LRRTM2 and NLGN1, for example, compete with each other for neurexin binding, though the functional consequences are not clear [56, 65, 66]. In contrast, at inhibitory synapses, neurexins form a* trans*-synaptic interaction with NLGN2 promoting inhibitory synapse development [23]. However, competing with this interaction, the postsynaptic adhesion molecule MDGA1 binds NLGN2 tightly side-by-side forming a* cis*-complex on the dendritic surface that prevents the neurexin:NLGN2* trans*-synaptic bridge, thereby decreasing inhibitory synapse development [16, 17] (Figure 4(c)). Also at inhibitory synapses, neurexin 1*α* binds to *α*-dystroglycan or neurexophilin 1 (NXPH1) in a mutually exclusive manner. One consequence of *α*-dystroglycan engaging the neurexin 1*α* L2 domain is that it prevents binding of neuroligins to the distant neurexin 1*α* L6 domain suggesting that an (allo)steric mechanism regulates these protein partner interactions [68], (refer back to Figure 1). Therefore, the neurexin-centered interactome provides examples of how SAMs can compete with each other for binding partners* in cis* or* in trans* and also be subject to (allo)steric mechanisms that regulate protein partner interactions.

It is possible that different SAMs interact with each other in a series of sequential and concerted steps to develop and regulate synapses; see also recent review by [69]. Revealing such a playbook of interactions will be no easy task because it is complex to accurately assess SAM function. Protein interactions that occur in vitro in a controlled experimental setting may not occur in vivo in the synaptic cleft or only under select circumstances. Likewise, SAM functions may exist in vivo that are not easily measurable in vitro. By way of illustration, neurexins are synaptogenic in vitro in coculture assays suggesting they are essential to form synapses, yet, in vivo, triple knockout of all three alpha- or beta-neurexins does not prevent synapse formation [70, 71]. Likewise, CNTNAP2, considered a* bona fide* SAM, does not appear important for synapse formation in and of itself, rather it prevents the elimination of new synapses in some way based on live imaging studies through cranial windows in mice [72]. Taken together, the broad portfolio of SAMs present in mammalian brain appears critical to generate diverse, adaptable protein interaction networks that mediate the different stages of a synapse, starting from its initial formation to its ultimate elimination, and to permit activity-dependent regulation once it has formed.

### 4.3. Diversification of SAMs through Alternative Splicing

One important mechanism to generate diversity of SAMs in the nervous system that deserves special attention is the process of alternative splicing, which has been shown to be regulated by synaptic activity in some cases (see (3) in Figure 3). Alternative splicing provides a very efficient and genetically “cost-effective” mechanism to generate a large panel of proteins that share a common scaffold but each differ from one another to some extent. Alternative splicing of mRNA transcripts result in insertions, deletions, and substitutions of amino acids in the encoded protein and can involve single residues, small inserts, or even complete domains. Several well-known SAM families undergo alternative splicing of their mRNAs generating a portfolio of protein molecules with altered properties and function.

Neurexins form one of the best studied families of SAMs diversified through alternative splicing. Neurexins are encoded by three genes (1, 2, and 3) that each produce a short beta form and a long alpha form, by virtue of two different promoters [23]; see also Figure 1. Single molecule mRNA sequencing of tens of thousands of neurexin mRNAs has demonstrated that there are at least ~1,400 variants by one report and more than 2,000 variants by another in the adult mouse brain [73, 74], though the transcripts are not all equally abundant [74]. Alternative splice inserts can be incorporated at six places in the extracellular region of neurexin 1*α* (SS#1 through SS#6), adding polypeptide inserts of up to 30 amino acids at five of these insertion sites; see Figure 1 and [23, 74]. Incorporation of splice inserts has functional consequences because several inserts have been shown to regulate the interaction of neurexins with different postsynaptic partners. For example, incorporation of SS#2 in the L2 domain of neurexin 1*α* decreases its binding to *α*-dystroglycan, while SS#4 regulates the affinity of neurexins to postsynaptic partners such as neuroligins, LRRTMs, *α*-dystroglycan, cerebellin precursor protein, and latrophilin/ADGRL (recently reviewed by [23, 68, 75]). Proteomic quantitation has confirmed that distinct neurexin splice variants bind different amounts of protein partners, corroborating a mechanism whereby alternative splicing regulates the binding affinity of neurexins for different ligands in vivo [76]. From a biochemical and protein structural perspective, SS#2 and SS#4 change the affinity of Ca^2+^-binding sites central to protein interaction sites on the L2 and L6 domains, while SS#4 also induces structural plasticity because it can adopt multiple conformations [77–79]. From a functional perspective, mice engineered to constitutively include SS#4 in neurexin 3*α* show a decrease in synaptic strength and impaired LTP in vivo because postsynaptic AMPA-receptor levels are decreased at the synapse (as a result of increased AMPA-receptor endocytosis), although the underlying mechanism is not clear [80]. For most neurexin splice inserts, however, their effects on protein structure and function are not well delineated. Likewise, the function of rare neurexin splice variants, in which multiple domains are deleted, is also not known, nor if these yield functional proteins in the first place [74].

The very large portfolio of neurexin alternative splice forms is strategically positioned to play an important role in synaptic plasticity. In the mammalian brain, specific neurexin splice forms demonstrate cell type specific distributions and brain region specific expression both at the mRNA as well as the protein levels [73, 76, 81]. Importantly, incorporation of certain splice inserts is neuronal activity dependent, and an altered splicing profile can be reversed [82–84]. For example, analysis of mRNAs in single medium spiny neuron cells (MSNs) demonstrated that neurexin 1*α* and neurexin 1*β* are prevalent in D_1_R-MSNs, but much less so in D_2_R-MSNs, and mostly contain the SS#4 insert [81]. However, exposure to repeated cocaine administration, a circumstance triggering synaptic plasticity, reduces neurexin 1 mRNA levels in D_2_R-MSNs even further and alters the profile of splice forms [81]. Therefore, alternative splicing of neurexins generates diversity of protein structure and function, and it can be regulated by events linked to synaptic plasticity.

Other SAM families are regulated by alternative splicing in their extracellular domain as well, altering the affinity with which they bind protein partners in the synaptic cleft. These include the neuroligins where splice inserts regulate interactions with neurexins (refer back to Figure 1, [85–88]); PTP*δ* and PTP*σ* where splicing regulates binding to Slitrks, interleukin-1 receptor accessory protein (IL1RAP), and SALM3 [89–94]; and the family of adhesion GPCRs where alternative splicing alters the domain composition of the extracellular region and consequently the profile of interacting protein partners [95].

### 4.4. Altered Location of SAMs within the Synaptic Cleft

The advent of powerful high resolution microscopy techniques has revealed that SAMs can be redistributed within the synaptic cleft in response to synaptic activity (see (4) in Figure 3). Recent studies show that the synaptic cleft is made up of structurally distinct subcompartments and SAMs can segregate to different regions of the cleft. Upon synaptic activity, however, certain molecules can move within or to the periphery of the synaptic cleft. The impact of these redistributions on synaptic function, however, is not clear. For instance, SynCAM1 and EphB2 receptor tyrosine kinase (EphB2) are two postsynaptic SAMs with different roles. SynCAM1 induces synapse formation and subsequently also maintains excitatory synapses, while EphB2 promotes excitatory synaptogenesis in the rapid early phase of synaptogenesis before neurons mature. By tracking SynCAM1 and EphB2 in the synaptic cleft at excitatory synapses, Perez de Arce and coworkers demonstrated that SynCAM1 is located around the cleft's edge while EphB2 is embedded deeper within the central PSD region [96]. Strikingly, upon application of an LTD paradigm, SynCAM1 underwent redistribution on the surface of the synaptic membrane forming puncta of increasing size, an intriguing finding given that SynCAM1 regulates LTD in vivo and suggesting this redistribution has functional significance [96]. Another SAM, N-cadherin, forms* trans*-synaptic bridges with N-cadherin molecules tethered to the opposing synaptic membrane. N-cadherin plays an important role presynaptically by regulating synaptic vesicle recruitment and recycling, and postsynaptically in spine remodeling and trafficking of AMPA-Rs, which is important for hippocampal LTP [97]. Superresolution microscopy has shown that N-cadherin localizes predominantly as puncta at the periphery of synapses and to a much lesser extent along the synaptic cleft in unstimulated cultured hippocampal neurons [97]. However, upon synaptic stimulation followed by a rest period, N-cadherin distributes broadly throughout the synaptic cleft [97]. Thus an increasing body of work shows that SAMs can be redistributed as a result of synaptic activity, likely altering protein interactomes in the synaptic cleft. How different SAMs are redistributed and the impact of such redistribution on synaptic function remain to be further elucidated.

### 4.5. Astrocytic Control of SAMs

A fascinating development has been the demonstration that astrocytes (a type of glial cell found interspersed between neurons which can ensheath synapses) secrete factors that modulate the action of SAMs (see (5) in Figure 3). During the development of the nervous system, astrocytes regulate synapse formation and remodeling, impacting synapse number through their ability to promote the formation and elimination of synapses [98]. A single mouse astrocyte can ensheath more than 100,000 synapses [99]. In the mature brain, astrocytes also can modulate synaptic plasticity [98]. Immature astrocytes secrete thrombospondin 1 and thrombospondin 2 (TSP-1 and TSP-2), large, trimeric extracellular matrix proteins that promote the formation of silent synapses in vitro and in vivo (i.e., synapses that are presynaptically active, but postsynaptically silent because they lack functional AMPA-Rs) [100]. TSP1 can bind postsynaptic neuroligins, increasing the speed of excitatory synapse formation at early stages in cultured rat hippocampal neurons, although not the final density of the synapses formed in mature neurons [101]. Hevin, another protein secreted by astrocytes, can modify the interaction between two SAMs in the synaptic cleft by working as an adaptor protein [102]. Hevin binds directly to neurexin 1*α* and NLGN1(+B), a pair of SAMs that normally do not interact, and engages them in a* trans*-synaptic bridge promoting excitatory synapse formation [102]. It is thought that the nine-amino-acid splice insert at site B in NLGN1(+B) sterically blocks the interaction between NLGN1 and the sixth LNS domain of neurexin 1*α* (L6) (refer back to Figure 1) thereby forming a key component of the “neurexin-neuroligin splice code,” reviewed in [23]. The bridging of neurexin 1*α* and NLGN1(+B) by hevin, overriding the splice code, was shown to be critical to form thalamocortical connections in the developing visual cortex in vivo [102]. Therefore, astrocytes by secreting proteins that interact with* bona fide* SAMs can modify their interactions and regulate protein interactomes in the synaptic cleft.

### 4.6. Novel Mechanisms to Regulate SAMs

It is likely that additional novel mechanisms exist that regulate SAMs, impacting their function in synaptic activity-dependent ways. One tantalizing mechanism is that SAMs undergo protein structural changes in response to synaptic activity. Perhaps mechanisms will be validated confirming that SAMs can sense synaptic activity in the synaptic cleft and adjust their protein interactions in response via (allo)steric mechanisms. Certainly, incorporation of an alternative splice insert in a SAM in response to synaptic activity (as discussed above) would be one way to induce a protein conformational change. Such a splice insert driven conformational change would have the potential to alter protein interactions within the synaptic cleft. The splice inserts SS#1 and SS#6 in neurexin 1*α* are of interest in this respect because they integrate into molecular hinges within the neurexin ectodomain and are poised to alter the conformation of domains with respect to one another. However, it is not known yet if these splice inserts are subject to activity-dependent incorporation [74, 103]. A novel protein conformation or interaction site in a SAM might also be induced upon binding of a protein partner and controlled though synaptic activity-induced expression of that partner (refer back to Figures 2(b) and 2(c)). Neuronal activity-induced expression of *α*-dystroglycan [40], which binds the L2 domain of neurexin 1*α* and appears to sterically block the interaction of neurexin 1*α* with neuroligins via the L6 domain, is a prime example [68] (refer back to Figure 1). Synaptic stimulation also appears to induce homodimerization of N-cadherin, an event altering the overall protein architecture [104]. Lastly, the protein conformation of a SAM containing Ca^2+^-binding sites might also be altered by changes in Ca^2+^ levels in the synaptic cleft as a result of synaptic activity, affecting its interactions with protein partners. Experimental evidence is accumulating that Ca^2+^ levels decrease in the synapse cleft in response to (prolonged) synaptic activity, a result of Ca^2+^ flooding into the presynaptic terminal during synaptic vesicle release and/or into the postsynaptic terminal upon NMDA-receptor activation [105, 106]. It has been suggested that the extracellular Ca^2+^-level in the synaptic cleft is ~1 mM and can drop significantly, maybe as much as 30–60% as presynaptic and postsynaptic channels open [107]. Studies on* trans*-complexes of cadherins have shown that their interactions depend in part on extracellular Ca^2+^ levels and are rapidly decreased when extracellular Ca^2+^ is depleted [108]. Thus, additional and novel mechanisms to regulate SAMs in response to synaptic activity may be validated in the near future.

## 5. SAMs Are Implicated in Neuropsychiatric and Neurodevelopmental Diseases

Many SAMs, including neurexins, neuroligins, LRRTMs, and other leucine rich repeat containing proteins, contactins, CNTNAPs, and cadherins are now implicated in neuropsychiatric and neurodevelopmental diseases, such as autism spectrum disorder, schizophrenia, bipolar disorder, epilepsy, and mental retardation [8, 20, 22, 24, 26, 109]. Initially, it was speculated that these molecules played crucial roles in the formation of synapses, and their lesion would lead to large scale disruption of synapse formation. Nevertheless, it was puzzling why deficits in such molecules, if indeed so essential for synapse formation, were selectively linked to cognitive and behavioral disorders, leaving other brain functions such as the coordination of movement or the processing of auditory and visual information apparently undisturbed. It is now recognized that there is a very large portfolio of SAMs in the mammalian brain, and there is not one single SAM, which when deleted, is sufficient to prevent synapse formation on a large scale given their partially redundant and overlapping functions. Furthermore, we now realize that the function of SAMs is much more complex and nuanced than purely the adhesion of presynaptic and postsynaptic membranes, as discussed in this review. It is also clear that various SAMs have discrete localization to very select groups of synaptic contacts, imparting their functional role in a synapse selective way. Recent work is focusing on unravelling the exact contribution of different SAM family members at specific synaptic contacts in order to understand how they mediate select neural circuits; see, for example, [110]. In addition, increasing attention is being paid to SAMs that selectively localize to excitatory or inhibitory synapses, respectively. An imbalance in excitatory (*E*) versus inhibitory (*I*) synaptic transmission has been speculated to play a role in the pathogenesis of neuropsychiatric disease, though whether this is a root cause or a result of other molecular processes that have been disrupted is not clear; for a recent review see [111]. Importantly, altering the level of select SAMs in animal models alters excitatory and/or inhibitory transmission (affecting the *E*/*I* balance) and leads in parallel to cognitive and social deficits, recently reviewed in [20, 112, 113]. In summary, it is still largely unknown how exactly the different SAMs contribute to the molecular mechanisms that underlie the pathogenesis of neuropsychiatric and neurodevelopmental diseases. Certainly, determining for each implicated SAM (1) at which synapses the SAM is present, (2) what its role is in developing, as well as mature brain, and (3) how the SAM is dynamically regulated will provide vital information to assess the role of that particular SAM in cognitive and behavioral disorders.

## 6. Conclusion

SAMs play a key role in establishing and maintaining synapses; they are involved in synapse formation, development, maturation, and elimination. Through their roles at synapses, SAMs are in position to impact the flow of information throughout the brain and beyond. Exciting work is being done to investigate the extent to which SAMs respond to synaptic activity modifying their protein interactions and function. Because SAMs are implicated in neuropsychiatric and neurodevelopmental disorders, studying their precise molecular mechanisms and interaction modes with their partners holds promise that this information can eventually be leveraged to design completely novel therapeutic strategies that regulate aberrant synaptic communication.
